# 

**Published:** 1923-10

**Authors:** 


### HOSPITAL and HEALTH

REVIEW

*Established 1886* *No.* 1867. *Vol. LXXII.*

*New Series.* Vol. II No. 25 *October,* 1923. Price Sixpence.

CONTENTS.

| | PAGE |
|---|---|
| The Ministry and its Ministers | 355 |
| Spiritual Healing | 356 |
| Notes and Comments | 357 |
| The Growth of Cancer—Still C3—Contributory Schemes—The Slum—Compulsory Vaccination—Too Dull or Too Clever?—Sir William Treloar—The Purity of Food—The Problem of the Diphtheria Carrier—Peopling the Dominions—Medical Tests for Drunken Drivers—The Blood-Pressure Hypochondriac—The Omnipotent Thyroid Gland—Healthier Womanhood—The Dreyer Tuberculosis Vaccine. | |
| The Public Health: Interviews with Local Authorities—The County of Leicester | 361 |
| Correspondence | 363 |
| Work of the Red Cross | 363 |
| Hospital Co-Operation | 364 |
| If the Doctors Refuse? | 365 |
| Rhyl Sanitary Congress | 366 |
| Contributory Schemes at Work | 367 |
| The Hartshill Orthopædic Hospital | 368 |
| National Health Insurance Notes | 369 |
| Hospitals and their Milk Supply | 370 |
| Responsibility for Dirty Milk | 372 |
| The National Health | 373 |
| A Dustless and Washable Hospital | 375 |
| The Hospital Needs of Swindon | 376 |
| Hospital Progress and Finance | 377 |
| Reviews of Books | 379 |
| Echoes of the Month | 383 |
| Hospital and Health News | 384 |
| Points from Hospital Reports | 386 |
| Recent Appointments | 386 |

